# Dual time point [^18^F]FLT-PET for differentiating proliferating tissues vs non-proliferating tissues

**DOI:** 10.1186/s13550-019-0579-5

**Published:** 2019-12-12

**Authors:** Pierre Lovinfosse, Caroline Rousseau, Jean-Yves Pierga, Francis Bouchet, Alexandre Cochet, Jean-Louis Alberini, Sylvie Girault, Pierre Vera, Pierre Olivier, Lionel Uwer, Florent Cachin, Benoit Scarwell, Jérome Lemonnier, Emmanuelle Fourme, Christel Mesleard, Anne-Laure Martin, Franck Lacœuille, Olivier-François Couturier

**Affiliations:** 1 Nuclear Medicine Department and Inserm UMR_S 1066 MINT, University of Angers, Angers, France; 2 Nuclear Medicine Department, West Cancer Institut (ICO), René Gauducheau Centre, Saint Herblain, France; 3 Nuclear Medicine Department, Curie Institut, Paris, France; 4 Nuclear Medicine Department, Georges-François Leclerc Centre, Dijon, France; 5 Nuclear Medicine Department, Curie Institut, Saint-Cloud, France; 6 Nuclear Medicine Department, West Cancer Institut (ICO), Paul Papin Centre, Angers, France; 7 Nuclear Medicine Department, Henri Becquerel Centre, Rouen, France; 8 Nuclear Medicine Department, University of Nancy, Nancy, France; 9 Nuclear Medicine Department, Institut de cancerologie de lorraine, Vandoeuvre-les-, Nancy, France; 10 Nuclear Medicine Department, Jean Perrin Center, Clermont Ferrand, France; 11 Nuclear Medicine Department, Centre Hospitalier de la Cote Basque, Bayonne, France; 12 UniCancer R&D, Paris, France

**Keywords:** Fluorothymidine, FLT, Positron emission tomography, Breast cancer

## Abstract

**Purpose:**

For differentiating tumor from inflammation and normal tissues, fluorodeoxyglucose ([^18^F]FDG) dual time point PET could be helpful. Albeit [^18^F]FLT is more specific for tumors than [^18^F]FDG; we explored the role of dual time point [^18^F]FLT-PET for discriminating benign from malignant tissues.

**Methods:**

Before any treatment, 85 womens with de novo unifocal breast cancer underwent three PET acquisitions at 33.94 ± 8.01 min (PET30), 61.45 ± 8.30 min (PET60), and 81.06 ± 12.12 min (PET80) after [^18^F]FLT injection. Semiquantitative analyses of [^18^F]FLT uptake (SUV) were carried out on tumors, liver, bone marrow (4th thoracic vertebra (T4) and humeral head), descending thoracic aorta, muscle (deltoid), and contralateral normal breast. Repeated measures ANOVA tests and Tukey’s posttests were used to compare SUVmax of each site at the three time points.

**Results:**

There was a significant increase in SUVmax over time for breast lesions (5.58 ± 3.80; 5.97 ± 4.56; 6.19 ± 4.42; *p* < 0.0001) (m ± SD for PET30, PET60, and PET80, respectively), and bone marrow (for T4, 8.21 ± 3.17, 9.64 ± 3.66, 10.85 ± 3.63, *p* < 0.0001; for humeral head, 3.36 ± 1.79, 3.87 ± 1.89, 4.39 ± 2.00, *p* < 0.0001). A significant decrease in SUVmax over time was observed for liver (6.79 ± 2.03; 6.24 ± 1.99; 5.57 ± 1.74; *p* < 0.0001), muscle (0.95 ± 0.28; 0.93 ± 0.29; 0.86 ± 0.20; *p* < 0.027), and aorta (1.18 ± 0.34; 1.01 ± 0.32; 0.97 ± 0.30; *p* < 0.0001). No significant difference was observed for SUVmax in contralateral breast (0.8364 ± 0.40; 0.78 ± 0.38; 0.80 ± 0.35).

**Conclusion:**

[^18^F]FLT-SUVmax increased between 30 and 80 min only in proliferating tissues. This could be helpful for discriminating between residual tumor and scar tissue.

## Introduction

Despite a lack of specificity for tumor, [^18^F]FDG remains the main tracer for positron emission tomography (PET). This success is explained by its high sensitivity and its capability to stage a wide variety of tumors. It has been shown that tissues with high levels of glycolysis will continue to accumulate over time [^18^F]FDG as [^18^F]FDG-6-phosphate [1, 2]. For differentiating malignant lesions from inflammation and normal tissues, a dual time point imaging protocol has therefore been proposed, since tumor [^18^F]FDG uptake may increase over time but remain unchanged or decreased in normal or inflammatory tissues. Another approach is to develop new PET tracers offering improved specificity for tumor such as proliferation marker, uncontrolled tumor proliferation being one of the fundamental characteristics of cancer [3].

[^18^F]Fluorothymidine ([^18^F]FLT) is the leader of fluorinated thymidine PET tracer, which tumor uptake is directly related to the thymidine kinase 1 (TK1) activity. [^18^F]FLT tumor uptake reflects tumor proliferation in most human cancers [3, 4] and is assumed to be more specific than [^18^F]FDG but less sensitive, related to high [^18^F]FLT activity in bone marrow and liver, limiting the detection of lesions in these common sites of metastases. In human breast cancer, high levels of TK1 activity has been observed [5], many fold higher than those of normal mammary tissues in particular in rapidly proliferating tissues such as breast adenocarcinoma [6–8]. 5' nucleotidase competes with TK1 and is responsible for the dephosphorylation of [^18^F]FLT monophosphate, but its activity is too low to balance phosphorylation of [^18^F]FLT. [^18^F]FLT undergoes two other phosphorylations but is slightly incorporated into DNA due to the stability conferred by the 18F-fluorination [9]. The consequence of these metabolic phenomena is an intracellular accumulation of [^18^F]FLT, comparable to that observed with [^18^F]FDG [10], and it can be hypothesized that [^18^F]FLT accumulates over time in tumors as seen with [^18^F]FDG.

There is no standardized protocol for performing [^18^F]FLT-PET scans, namely, no standardization for the uptake period. Kenny et al in a small cohort of 15 breast cancer patients reported that the values of semiquantitative parameters evaluating [^18^F]FLT uptake were similar when monitored early (21 min) or late (90 min) post-[^18^F]FLT injection [11]. Likewise, using a 45-min dynamic sequence immediately after intravenously administration of [^18^F]FLT, Pio et al. showed that tumor uptake from the 5- to 10-min first frames was able to predict longer-term clinical outcome [12]. Recently, Zhang et al. reported that e 16-min dynamic PET acquisition appears to be sufficient to provide accurate [^18^F]FLT kinetics to quantitatively assess the proliferation in breast cancer lesions [13]. Despite the lack of consensus, most studies use the same settings than those of [^18^F]FDG procedures, i.e., a period of 60 ± 10 min of uptake before scanning [7, 11, 14–24]. In the present study, using a multiple-point acquisition protocol, we aimed to study the behavior of [^18^F]FLT uptake over time in different tissues in women with a de novo locally advanced breast cancer prior the beginning of neoadjuvant anthracycline-based chemotherapy.

## Material and methods

### Patients

Between February 2007 and March 2012, 122 women were included in this multicentre FLT01 study (NCT 00534274, 13 centers, France). Inclusion criteria were non-operable unifocal T2 or T3 breast tumor, M0 whatever N, histologically proven (all types except lobular invasive grade I), not overexpressing c-erbB2 thus eligible for a neoadjuvant anthracycline-based chemotherapy. Noninclusion criteria were multifocal and/or bilateral breast tumor, metastatic invasion except lymph node involvement, inflammatory breast tumors, patient already included in another trial with experimental therapeutic molecule, pregnancy, woman deprived of liberty or under guardianship, inability to submit follow-up medical testing.

Eighty-five women (age : 49.39 ± 10.30 years (m ± DS); bodyweight: 67.49 ± 13.56 and BMI : 25.47 ± 4.58) had a complete three-point acquisition protocol at baseline [^18^F]FLT-PET, i.e., before the beginning of the neoadjuvant chemotherapy.

This study was approved by the ethics committee of the University Hospital of Angers, and all patients signed their informed consent before enrollment.

### Histology

Three 14 G (or 16 G) micro-biopsies, one of which was frozen, were performed under ultrasound control for each tumor, with histological and histopronotic grade determination according to modified Scarff-Bloom-Richardson. Histological analysis of these 85 tumors revealed 73 ductal carcinomas, 10 lobular carcinomas, 1 metaplastic carcinoma, and 1 adenoid cystic carcinoma, among which 4 SBR I, 46 SBR II, 34 SBR III, and 1 indeterminable SBR grade (Table 1).

**Table 1:** Tumor characteristics

Tumors	Total	SBR I	SBR II	SBR III	Indeterminable SBR
*Ductal carcinoma*	73	4	36	32	1
*Lobular carcinoma*	10	0	9	1	0
*Metaplastic carcinoma*	1	0	0	1	0
*Adenoid cystic carcinoma*	1	0	1	0	0
*Total*	85	4	46	34	1

### Study design

Before inclusion, a conventional staging was performed in all patients, i.e., clinical examination, bilateral mammogram, breast ultrasound, and echo-guided biopsy for histological analysis. The patients were included if they met all inclusion criteria without any noninclusion criteria. The [^18^F]FLT-PET examination was performed away from the biopsy (at least 10 days) but as soon as possible thereafter, not to delay the onset of the neoadjuvant chemotherapy.

### Combined [^18^F]FLT-PET/CT studies

The same acquisition [^18^F]FLT-PET procedure was followed by all the 13 centers.

Patients were not fasting but well oral hydrated. A venous catheter with an infusion of 500 mL of saline serum was placed in a peripheral vein (in the contralateral arm to the breast tumor). According to the type of [^18^F]FDG-PET scanner used, a [^18^F]FLT dose of about 3 MBq/kg (3D mode) or about 5 MBq/kg (2D mode) body weight was injected (mean ± SD, 4.54 ± 0.81 MBq/kg; min–max, 2.85–6.41 MBq). The mean ± SD-injected dose was 305.53 ± 77.68 MBq (min–max: 137–495 MBq).

After an uptake phase of 20 min, the patient was set in prone position in the PET machine, and the first PET acquisitions (PET30) focused on breasts and axillae which started immediately (uptake times; m ± SD: 33.94 ± 8.01 min.). For all series of PET/CT images, a low-dose CT scans were acquired first (120 to 140 keV; 80 to 100 mAs, depending on the routine procedure of each center), followed by PET scans with an acquisition time of 5 min per bed position. At the end of PET30 acquisitions, the patient remained in the PET system until the start of the second PET (PET60) acquisitions focused on breasts and axillae with exactly the same settings (uptake time; m ± SD: 61.45 ± 8.30 min.). Thereafter, the patient was set in supine position for the whole body PET scan (PET80) (pelvis to base of the skull, uptake phase (uptake times; m ± SD: 81.06 ± 12.12 min)).

The CT data were used for attenuation correction, and [^18^F]FLT-PET images were reconstructed by using a standard iterative algorithm depending on each type of PET machine (ordered subset expectation maximization or row-action maximalization-likelihood iterative algorithm). A centralized reading was performed with a dedicated software providing multiplanar reformatted images of non-attenuated PET, attenuated PET, CT, and fused data with linked cursors (IMAGYS® Workstation, 2010 – Kéosys, St Herblain, France).

### [^18^F]FLT-PET data analysis

Semiquantitative analysis of [^18^F]FLT uptake has been carried out using standardized uptake values (SUV) normalized by body weight.

Differents types of SUVs were computed for breast tumors: maximum SUV (SUVmax; maximum pixel value), mean SUV (SUV41; computed on a 3D isocontour at 41% of the maximum pixel value), and peak-equivalent SUVs (SUVpeak computed on a 3D ROI of 1 cm^3^ containing the maximum pixel value but not necessarily centered by the maximum pixel value).

For normal tissues, SUVmax and SUVmean were calculated on the liver (3D ROI of 3 cm diameter drawn on the right liver area following PERCIST recommendations [25]) and on a 3D ROI of 1 cm diameter for the others tissues, i.e., contralateral breast, descending aorta, muscle (deltoid), and bone marrow (humeral head and T4 vertebral body).

### Statistical analysis

Comparisons of [^18^F]FLT SUV in normal tissues and tumors, i.e., between PET30, PET60, and PET80 data were carried out by using variance analysis tests (repeated measures one-way ANOVA). These analyses were done for each type of SUV and SUV ratio. Tukey’s multiple comparison posttests were used for identifying the significant differences between two sets of data.

About 0.05 was set at the level of statistical significance.

All statistical tests were performed with Prism® 4 for Macintosh, version 6.0 h, 2015, Graphpad® software Inc., La Jolla, CA 92037 USA)

## Results

SUVmax was chosen as the parameter of reference for describing the results of the present study. Endeed, SUVmax is the most documented semiquantitavive PET parameter in the literature, and furtheremore in this study, SUVpeak and SUVmean values followed most often the same profiles as SUV max values (in case of different behaviors between the different type of SUV, these differences have been reported).

### Tumor characteristics

Histological analysis of these 85 tumors revealed 73 ductal carcinomas, 10 lobular carcinomas, 1 metaplastic carcinoma, and 1 adenoid cystic carcinoma, among which four SBR I, 46 SBR II, 34 SBR III, and 1 indeterminable SBR grade (Table 1).

### Biodistribution of [^18^F]FLT in normal tissues

Whatever the type of SUV, at all acquisition times (i.e., PET30, PET60, and PET80), vertebral bone marrow was the tissue with the most important [^18^F]FLT uptake, followed by respectively liver and humeral head [^18^F]FLT uptakes (SUVmax values are summarized in Table 2). In contrast, normal breast tissues (contralateral to the primary breast tumor), thoracic aorta, and muscle showed the weakest [^18^F]FLT uptake.

**Table 2:** SUVmax of different normal target tissues over time

SUV max	PET30 m ± SD (min–max)	PET60 m ± SD (min–max)	PET80 m ± SD (min–max)
Contralateral normal Breast	0.83 ± 0.40 (0.24–2.12)	0.78 ± 0.38 (0.21–2.35)	0.80 ± 0.36 (0.25–2.48)
Muscle	0.95 ± 0.28^*^ (0.52–1.75)	0.93 ± 0.29 (0.37–1.65)	0.86 ± 0.20^*^ (0.32–1.21)
Descending aorta	*1.18 ± 0.34*^*††*^ (0.38–2.03)	*1.01 ± 0.32*^*†*^ (0.45–1.85)	*0.97 ± 0.30*^*†*^ (0.38–2.46)
Humeral head	*3.36 ± 1.79*^*‡‡*^ (0.78–10.74)	*3.87 ± 1.89*^*‡‡*^ (0.64–9.95)	*4.39 ± 2.00*^*‡‡*^ (0.56–9.57)
Liver	*6.79 ± 2.03*^*††*^ (2.96–12.65)	*6.24 ± 1.99*^*††*^ (2.92–12.84)	*5.57 ± 1.74*^*††*^ (2.41–9.81)
Vertebral body	*8.21 ± 3.17*^*‡‡*^ (2.13–18.8)	*9.64 ± 3.66*^*‡‡*^ (3.37–21.57)	*10.85 ± 3.63*^*‡‡*^ (3.72–20.72)

*significant decrease; *p* < 0.05

†significant decrease; *p* < 0.0001

‡significant increase; *p* < 0.0001

Normal breast tissues [^18^F]FLT uptake did not significantly vary over time, and only a weak decrease in [^18^F]FLT uptake between PET30 and PET80 was observed for muscle. Probably due to the elimination and metabolism of [^18^F]FLT, a significant decrease in [^18^F]FLT uptake was observed between all acquisition times in aorta (repeated measures ANOVA *p* < 0.0001) and in liver (*p* < 0.0001). In three cases (3, 5%), an intense activity was observed in the gall bladder, and in one of these three patients, the main bile duct was also visualized. After glucuronidation of [^18^F]FLT in the liver, the metabolites are excreted in urine which explains a bladder activity in all patients. In 17 patients (20%), a thymic uptake was observed.

The bone marrow of the 4th dorsal (D4) vertebral body was the normal tissue displaying the most important [^18^F]FLT uptake and increase (*p* < 0.0001) in tracer uptake over time. The most important variation in D4 uptake was seen between PET30 and PET80 (SUVmax average increase of 2.64 (+ 32%) ± 1.45). The humeral head [^18^F]FLT uptake followed the same variations with a significant increase over time (the most important SUVmax average increase was observed between PET30 and PET80 with a value of 2.64 (+ 31%) ± 1.45).

### Tumor uptake of [^18^f]FLT

**Fig. 1:**
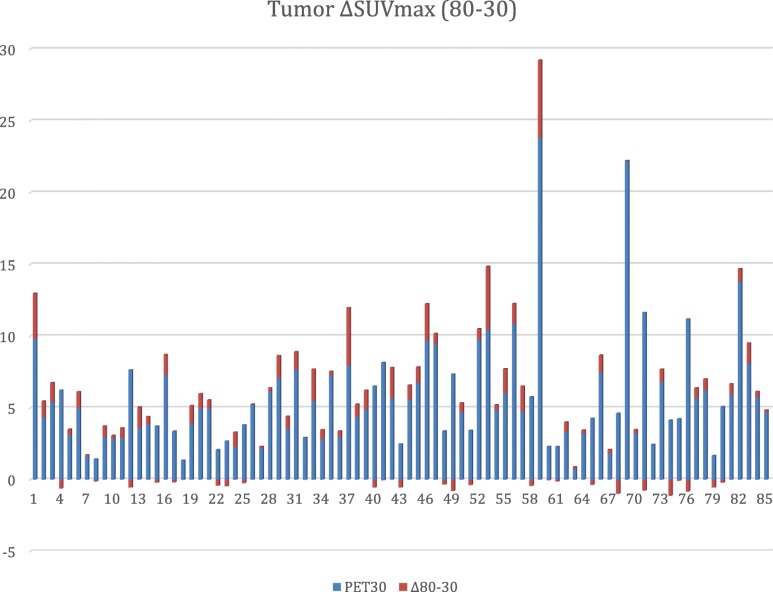
Primary breast tumor SUVmax differences between PET80 and PET30 (∆SUVmax). ∆SUVmax is plotted vs PET30 SUVmax. Note that 24 patients showed a decrease in FLT uptake, even though these decreases are marginal or of low amplitude. However, 50% of the patients correspond to an increase in FLT uptake between 30 and 60 min (79% are ductal carcinoma, and 58% are SBR II)

Table 3 and Fig. 1 summarize the tumor uptake values. Breast tumor [^18^F]FLT uptake increased significantly over time (repeated measures ANOVA *p* < 0.0001). The most important increase was observed between PET30 and PET80 (SUVmax average increase of 0.61 (+ 11%) ± 1.13).

**Table 3:** Primary breast tumor SUV over time

Breast Tumor	PET30 m ± SD (min–max)	PET60 m ± SD (min–max)	PET80 m ± SD (min–max)
SUVmax	*5.58 ± 3.80*^*†‡*^ (0.73–23.84)	*5.97 ± 4.56*^†*^ (0.75–29.28)	*6.19 ± 4.42*^‡*^ (0.88–29.22)
SUVpeak	*4.27 ± 2.63*^*†‡*^ (0.66–16.59)	*4.49 ± 3.10*^†*^ (0.63–19.63)	*4.66 ± 3.10*^‡*^ (0.71–18.61)
SUVmean41%	3.38 ± 2.27^*‡^ (0.58–14.35)	3.55 ± 2.66^**^ (0.53–17.32)	3.78 ± 2.70^*‡^ (0.54–16.86)

*significant increase; *p* < 0.05

†significant increase; *p* < 0.001

‡significant increase; *p* < 0.0001

All the 85 primitive breast tumors were clearly visualized at all acquisition times. The ratios between tumor [^18^F]FLT uptake and normal tissues (contralateral normal breast, aorta, liver, and muscle) were measured for each acquisition time (Table 4). The highest ratios were observed between tumor and normal breast tissues for PET60 whereas tumor to liver ratios were the smallest whatever the acquisition time. All these ratios increased significantly over time between PET30 and PET60 and PET30 and PET80 (repeated measures ANOVA *p* < 0.0001). The most important increase over time was observed for tumor to muscle ratios between P30 and P80 (SUVmax average increase of 3.04 (+ 57%) ± 2.90).

**Table 4:** Primary breast tumor to background SUVmax ratio

SUV max ratio	PET30 m ± SD (min–max)	PET60 m ± SD (min–max)	PET80 m ± SD (min–max)
Primitive Tumor to contralateral Breast	*7.94 ± 5.83*^*†‡*^ (1.16–32.66)	*9.05 ± 6.72*^*†*^ (1.38–40.11)	*9.00 ± 6.24*^*‡*^ (1.5–33.98)
Primitive Tumor to descending Aorta	*4.88 ± 2.75*^*‡‡*^ (0.62–16.22)	*6.10 ± 4.04*^*‡*^ (0.86–25.46)	*6.66 ± 4.74*^*‡*^ (0.88–34.79)
Primitive Tumor to Liver	*0.84 ± 0.46*^*‡‡*^ (0.13–2.40)	*0.98 ± 0.59*^*‡‡*^ (0.10–2.97)	*1.18 ± 0.75*^*‡‡*^ (0.12–3.56)
Primitive Tumor to Muscle	*5.29 ± 3.09*^*‡‡*^ (0.75–21.10)	*6.71 ± 4.56*^*‡‡*^ (0.81–32.53)	*8.33 ± 5.15*^*‡‡*^ (1.28–31.09)

†significant increase; *p* ≤ 0.001

‡significant increase; *p* ≤ 0.0001

## Discussion

The concept of dual time point (DTP) has been the subject of numerous publications with [^18^F]FDG in various cancer types. It has been shown that tissues with high levels of glycolysis will continue to accumulate [^18^F]FDG as [^18^F]FDG-6-phosphate, whereas tissues with high glucose-6-phosphatase activity (such as liver) will peak early, followed by a decrease in [^18^F]FDG retention [1, 2]. Studies on the DTP concept were conducted to define the optimal time needed between tracer injection and image acquisitions to distinguish between benign and malignant lesions.

The first study was conducted in head and neck tumor patients in 1999 by Hustinx R. et al. [26] and showed an increase in [^18^F]FDG tumor uptake between two acquisitions performed with 28 min of mean interval (70 and 98 min). The use of DTP in lung lesions showed conflicting results, but overall it seems that DTP increases the sensitivity in detecting the malignancy of lesions while the specificity remains stable or even decreases [27–33]. One of the main problems pointed out in theses studies is the lack of consensus to define a threshold of [^18^F]FDG uptake for malignancy.

In 1999, Boerner et al. demonstrated the superiority of a 180-min image acquisition in the detection of breast cancers and metastatic lymph node lesions compared to 40-min and 90-min acquisitions. In 2005, Kumar et al. reported a significant increase in SUV between the two acquisitions of DTP (ΔSUV) for breast cancer lesions [34]. The effectiveness of ΔSUV was reported for the detection of small cancers located in dense breasts (120) and for the determination of malignancy in breast lesions with a low initial uptake (SUVmax ≤ 2.5) [35–37]. However, for the detection of metastatic axillary lymph nodes, DTP may not increase the overall performance of [^18^F]FDG-PET, due in particular to false positives at the late time [38, 39].

The main problem of DTP studies is the lack of methodological homogeneity, leading to conflicting results [2]. Several factors could be identified: (1) different delays of late acquisitions, with sometimes an overlap between early and late acquisitions coming from different studies (major source of conflicting results); (2) different gold standards or criteria for the determination of malignancy (SUVmax threshold, retention index); (3) different study designs (pre- or post-therapeutic evaluation, patient population size and characteristics, various cancer types, etc.). However, studies agree on the following points: (1) background noise generally decreases on late images leading to a better image quality; (2) uptake increases on late images for the majority of [^18^F]FDG-avid tumors; (3) better diagnostic performance compared to a single acquisition in most studies, i.e., a better sensitivity of lesion detection thanks to a better lesion/background ratio with a gain of specificity; (4) SUVmax that is dependent of multiple factors should not be used as the sole criterion but also retention index or a combination of SUV and retention index. Finally, the main limitation of performing DTP examinations is the difficulty of integrating this technique for all patients into a daily schedule of Nuclear Medicine services.

In our study, bone marrow and liver displayed the highest [^18^F]FLT uptake in all patients. The intense bone marrow uptake is related to its important proliferative activity and to the absence of [^18^F]FLT catabolism by thymidine phosphorylase, due to the 18F labeling at the 3' position of the thymidine which confers the stability to the molecule [40–42]. The intense liver activity is related to the hepatic glucuronidation of the molecule, observed only in primates and humans, who have an enterohepatic cycle. [^18^F]FLT metabolites are excreted in the bile (in our study, three patients had intense gallbladder activity) and reabsorbed by the small intestine, before its urine elimination [4, 41]. By contrast, controllateral breast and muscle tissues displayed the lowest FLT uptake due to their low cell proliferation.

All breast tumors were well individualized at all the three examination times, due to the high cancer uptake and/or the low uptake of surrounding tissues, confirming the results of previous studies with [^18^F]FLT in breast cancer patients [7, 12, 14, 43]. The highest uptake ratios were observed between breast tumor and normal breast tissues for PET60. We found also a significant increase over time in breast cancer [^18^F]FLT uptake. The most important increase over time was observed for tumor to muscle ratios between P30 and P80 (SUVmax average increase of 3.04 (+ 57%) ± 2.90). To our knowledge, there is currently no publication specifically dedicated to [^18^F]FLT DTP in the evaluation of cancers. As for [^18^F]FDG, there is a cellular trapping of [^18^F]FLT by proliferative cells that overexpress “es” transporters allowing [^18^F]FLT entry into the cell [44] and with a high TK1 activity that phosphorylates [^18^F]FLT [9, 44–47]. This process competes with 5'-nucleotidase dephosphorylation, which is largely minority compared to TK1 activity [10], and phosphorylated [^18^F]FLT is not incorporated into DNA [9, 48–50], both explaining intracellular [^18^F]FLT accumulation over time. In comparison, the different “non-proliferating” tissues (aorta, muscle, normal breast, and liver), with a low level of cells at the end of the G1 and S phase, do not overexpress “es” transporters [51] or do not exhibit TK1 activity and therefore present a decrease in [^18^F]FLT uptake over time. This explains also the significant increase in tumor to not tumor ratios and a better tumor contrast over time.

Although [^18^F]FLT is assumed to be more specific for tumor than [^18^F]FDG, the increase in [^18^F]FLT tumor uptake over time could play an important role in assessing the neoplastic character of equivocal breast lesion and internal lymph node lesions. In addition, while intense activity in the hepatic parenchyma decreases significantly over time, the increase in tumoral uptake over time may allow the detection of metastatic liver lesions, using preferably an interval of approximately 1 h between the early and late acquisitions, this interval showing the most significant differences in our study.

## Conclusion

[^18^F]FLT uptake of neoplastic and non-neoplastic proliferating tissues increases significantly when the time between tracer injection and image acquisition increases, while this increase is not observed for “non-proliferating” tissues. This observation may be useful for distinguishing between tumor residues and scar tissues, particularly when [^18^F]FLT uptake becomes weak after treatment. In addition, changes in [^18^F]FLT uptake over time underscore the importance of a rigorous methodology when patients are imaged many times, for instance, when evaluating response to therapy. To improve the assessment of tumor proliferation, dynamic [^18^F]FLT studies have already been used, but the dual time point may allow the whole or partial body assessment instead of just a single dynamic volume.

## Data Availability

The datasets generated during and/or analysed during the current study are available from the Sponsor UNICANCER on reasonable request. The French Breast Cancer Intergroup (UCBG) scientific board must study and validate all proposals that will require access to data.
